# Real-Time Parallel-Serial LiDAR-Based Localization Algorithm with Centimeter Accuracy for GPS-Denied Environments

**DOI:** 10.3390/s20247123

**Published:** 2020-12-11

**Authors:** Jakub Niedzwiedzki, Adam Niewola, Piotr Lipinski, Piotr Swaczyna, Aleksander Bobinski, Pawel Poryzala, Leszek Podsedkowski

**Affiliations:** 1 Institute of Machine Tools and Production Engineering, Lodz University of Technology, ul. Stefanowskiego 1/15, 90-924 Lodz, Poland; jakub.niedzwiedzki@dokt.p.lodz.pl (J.N.); adam.niewola@p.lodz.pl (A.N.); pawel.swaczyna@p.lodz.pl (P.S.); 203139@edu.p.lodz.pl (A.B.); leszek.podsedkowski@p.lodz.pl (L.P.); 2 Institute of Information Technology, Lodz University of Technology, ul. Wolczanska 215, 90-924 Lodz, Poland; 3 Institute of Electronics, Lodz University of Technology, ul. Wolczanska 211/215, 93-005 Lodz, Poland; pawel.poryzala@p.lodz.pl

**Keywords:** CUDA, GPGPU, Kalman filters, LiDAR localization, mobile robots, parallel processing

## Abstract

In this paper, we introduce a real-time parallel-serial algorithm for autonomous robot positioning for GPS-denied, dark environments, such as caves and mine galleries. To achieve a good complexity-accuracy trade-off, we fuse data from light detection and ranging (LiDAR) and an inertial measurement unit (IMU). The proposed algorithm’s main novelty is that, unlike in most algorithms, we apply an extended Kalman filter (EKF) to each LiDAR scan point and calculate the location relative to a triangular mesh. We also introduce three implementations of the algorithm: serial, parallel, and parallel-serial. The first implementation verifies the correctness of our innovative approach, but is too slow for real-time execution. The second approach implements a well-known parallel data fusion approach, but is still too slow for our application. The third and final implementation of the presented algorithm along with the state-of-the-art GPU data structures achieves real-time performance. According to our experimental findings, our algorithm outperforms the reference Gaussian mixture model (GMM) localization algorithm in terms of accuracy by a factor of two.

## 1. Introduction

Fast and precise robot localization is a fundamental problem in autonomous robotic systems. In most cases, localization is accomplished through the use of GPS-based devices [1]. Unfortunately, GPS localization requires an unobstructed line of sight to at least four GPS satellites. As a result, it cannot be used in many areas, which are called GPS-denied environments. Such areas are very common, to name only: building interiors, mine galleries, caves, tunnels, underwater, etc. Here we focus on localization in caves and mine galleries as we design an underground robot for the mining industry. Localization in such environments is still an open and challenging problem that is of interest to many leading scientific centers, e.g., DARPA, which announced the SubT Challenge—a competition for autonomous underground vehicles—in 2018 [2].

### 1.1. State of the Art

Caves and mine galleries, which are considered in this paper, are undoubtedly GPS-denied environments. In such an environment, alternative localization techniques must be used, such as accelerometer- and gyroscope-based dead reckoning [3], magnetic-field-based localization [4], ultrasonic-sensor-based localization [5], visual-based localization [6] or radio-signal-based localization. Radio-signal-based localization can take advantage of various well-known standards, such as Bluetooth [7], ZigBee [8], Wi-Fi [9], frequency modulation (FM) radio [10] or ultra-wideband (UWB) radio [11]. Such techniques use radio waves’ specific properties, such as the time difference of arrival of radio signals or the received signal strength indicator (RSSI), to calculate an object’s position [12]. The main drawback of radio-based localization systems is that they require anchors with known absolute locations to calculate a robot’s location [13], thus making their scope of application in caves and mine galleries very narrow. Another class of localization techniques relies on analyzing the images from RGB or RGB-D cameras mounted on a robot to find the robot’s location relative to surrounding objects [14,15]. Other techniques use light detection and ranging (LiDAR)-based distance measurements [16]. Such systems are commercially available, such as for example Hovermap by Emesent Pty Ltd., but their technical details are not disclosed. There are also open-source systems, such as Hector SLAM [17], LOAM [18], Cartographer [19], NDT [20], but their localization error accuracy is equal to several centimeters at best [21,22]. Yet another LiDAR-based localization systems are used in vehicle localization, e.g., [23], but their accuracy is still above 10 cm at best. Accurate localization is an important parameter for camera pose estimation relatively to LiDAR point scan [24], but even state of the art algorithms [25] guarantee around 0.5 m accuracy. Centimeter-accuracy LiDAR-based localization systems described in [26,27,28,29] are available, but the centimeter accuracy is achieved in 1D or 3D space not 6D space, as in our case. The above-mentioned techniques can also be fused to achieve improved localization accuracy by applying Kalman filters (KFs) [30], extended Kalman filters (EKFs), particle filters (PFs), unscented Kalman filters (UKFs) and other fusion [31] and adaptive [32] algorithms. For real-time localization in dark caves and mine galleries, the EKF-based fusion of accelerometer- and gyroscope-based dead reckoning with LiDAR-based distance measurements offers the best performance because these two techniques do not require good lighting conditions [33] nor anchors, and radio signals are strongly attenuated underground [34]. In this paper, we use an EKF for data fusion because the considered model is nonlinear. An EKF also yields more precise localization results than a PF does [35,36]. We do not use a UKF because, in our algorithm, the EKF is applied at each measurement point, meaning that the time-step interval is minimal, but the density of the acquired point cloud is relatively low. In such a case, the calculation of sigma points may cause the accuracy of the localization algorithm to deteriorate and unnecessarily increase the computational workload [37,38]. Caves and mine galleries have very specific 3-dimensional, tube-like, irregular shapes. Such shapes can only be effectively represented using 3D models [39], as it is impossible to reflect all the necessary details of such an environment using merely 2D and 2.5D maps, which are used in many terrestrial applications [40]. Unfortunately, the precise representation of 3D models using point clouds is a highly resource-consuming proposition, especially for real-time applications [41]. We use a triangular-mesh-based map representation and localize our robot relative to this triangular mesh map because it allows for much more compact representation without significant loss of accuracy [42,43], is more effective for vehicle navigation than other map representations [44] such as Elevation Mapping [45], OctoMap [46] or VoxBlox [47] and is more convenient for texture mapping [48]. Using a triangular mesh as a reference map for localization is also convenient because such a mesh can be created using out-of-the-box point cloud triangulation [49,50] and requires no further preprocessing.

Although localization algorithms using data from LiDAR scanners and inertial measurement units (IMUs) have been intensively studied over the last decade for many applications, there is surprisingly little literature concerning localization relative to triangular mesh maps. The majority of LiDAR-based localization algorithms use landmarks or local object features to localize a robot on a map [51,52,53]. However, this approach cannot be adapted to mine galleries and caves because such landmarks are challenging to identify in these environments, especially in long mine galleries and cave corridors. In such uniform surroundings, localization at the object level loses precision and leads to information loss. Other approaches involve the use of

- inertial sensors and a digital map of the mine [54];
- PF-based fusion of localization data from inertial sensors, gyroscopes, speed sensors and ultrasonic sensors [55];
- beacon tags [56,57];
- a neural network that takes a video stream as input [58];
- a combination of low-cost sensors, namely a UWB sensor, a beacon, an IMU and a magnetic field sensor, using Wi-Fi signals [59]; or
- IMU data, LiDAR data and color camera images [60].

However, none of these techniques permit a robot’s localization relative to a triangular mesh with centimeter accuracy. Most work on 3D LiDAR systems has focused on scan registration, which is used to estimate the relative transformation between two scans (i.e., the rigid-body transformation matrix, representing translation and rotation). Extensive research has focused on the iterative closest point (ICP) method and its variants [61]. The standard ICP method iterates over the nearest point pairs and optimizes the solution by minimizing the sum of the squares of the point-to-point Euclidean distances. The performance of the ICP method is mainly influenced by factors such as the environment type, the motion trajectory, the uncertainty of the initial pose, and the point cloud distortion caused by the motion of the robot. This method is sensitive to the accuracy of the initial value and shows poor adaptability to dynamic environments. Not only point-to-point correspondences but also point-to-line correspondences, point-to-plane correspondences, normal vectors, and curvatures can be used [62]. The point cloud distortion caused by robot motion can be mitigated by applying an advanced odometry system, e.g., using encoder measurements, visual odometry or IMU. Among these three techniques, LiDAR odometry seems to be the most robust approach, providing low drift. The state-of-the-art method called LiDAR odometry and mapping (LOAM) [18] uses edge points and planar points as features to compute point-to-line and point-to-plane distances. Recently, a lightweight and ground-optimized version called LeGO-LOAM [63] was proposed, which achieved an accuracy similar to that of LOAM with a reduced computational cost. To eliminate pose estimation error in LiDAR odometry, it is possible to extend the LOAM/LeGO-LOAM algorithm by integrating pose-graph-based simultaneous localization and mapping (SLAM) into the system [63]. Another recent approach for improving the localization accuracy achieved using LiDAR and IMU data involves applying extended and unbiased finite-impulse response filters in Kalman filtering [64]. This algorithm achieves approximately 20 cm accuracy even though localization is performed relative to flat surfaces. Another recent article addressing the problem of localization in mines was published by Li et al. [65]. The authors of this article used a normally distributed transform based on pose graph optimization and loop closure for SLAM localization. They also extracted planar surfaces to serve as landmarks. However, that article focused on SLAM rather than on localization relative to a known map and therefore achieved a much lower localization accuracy than that reported in [64]. To our knowledge, there are no algorithms that can be directly used to localize a robot relative to a triangular mesh map in a GPS-denied environment that can be applied in caves and mine galleries. In this article, we introduce a new algorithm that allows us to accomplish this task. To achieve real-time performance that is to be able to compute the robot localization with centimeter accuracy when it is moving with its maximal velocity of 2 m/s, we also introduce a parallel-serial version of this algorithm, which offers better performance in terms of time complexity.

The algorithm introduced in this article extends the concept of the LiDAR localization scheme introduced in [66], which performs well on long roads, where the risk that the longitudinal uncertainty will grow to infinity is high. An analogous problem arises in long caves and mine galleries. The localization algorithm presented in [66] uses fused data from a LiDAR scanner and an IMU, which is also our choice of data sources for a GPS-denied environment. To accelerate the localization process, the authors of that article discretized the reference point cloud onto a 2D grid, with each cell in the grid containing a Gaussian mixture characterizing the 3D points contained in the corresponding infinite column. Because a 2D map is sub-optimal for representing a cave or mine gallery, we use a similar approach but replace the 2D map with a 3D triangular mesh. Instead of using infinite columns projected onto a 2D plane, we use triangles that store the covariances obtained from reference LiDAR scans of the cave or mine gallery. To achieve low computational complexity, the localization algorithm introduced here uses the concept presented in [67], in which the pose correction for the robot is computed for each LiDAR measurement point separately. This reduces the computational complexity by eliminating the need for a time-consuming comparison of point clouds. However, unlike in [67], in our algorithm, the location is computed relative to triangles rather than surfels, which unfortunately makes the algorithm more demanding, as finding the closest triangle is a more resource-consuming task than finding a surfel on a 2D plane. Therefore, in Section 2.1, we introduce a parallel-serial version of the proposed localization algorithm to accelerate the calculations. Our algorithm outperforms the state-of-the-art Gaussian mixture map algorithm introduced in [66] because the Gaussian mixture algorithm is applied in only one dimension. As a result, the 3D representation obtained using the Gaussian mixture algorithm is a less precise representation of the reference point cloud than a triangular mesh of the same size is for an environment such as a cave or mine gallery. We do not compare our algorithm with algorithms that use full point clouds because the computational complexity of such algorithms is too high for the embedded, low-power-consumption, real-time hardware that is usually used on small mobile robots that can operate in mine galleries and caves.

Additionally, in full point cloud methods, robot motion gives rise to a phenomenon called point cloud distortion, which increases the localization error. In our method, in which a position correction is calculated after each measurement, this source of error is eliminated, thus improving the accuracy.

### 1.2. Novelty

The key contributions of this paper include the following:

- a novel localization algorithm in which a 3D triangular mesh map is used as the reference for localization,
- robot pose correction calculations for each LiDAR measurement in the triangular mesh,
- serial and parallel-serial implementations of the algorithm, and
- an evaluation of the proposed algorithm on data obtained from cave and mine gallery environments.

The rest of this paper is organized as follows. First, we present a general outline of the proposed localization algorithm. Then, we describe the individual steps of the localization algorithm, namely the map search and EKF procedures, as well as its serial and parallel-serial implementations. We then describe the environments used for testing and present corresponding measurement results. Finally, we discuss those results.

## 2. Material and Methods

### 2.1. Localization

**Figure 1:**
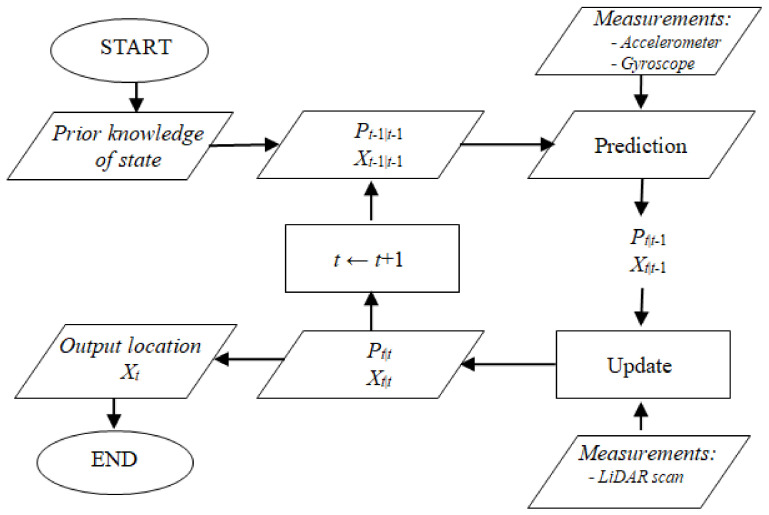
Outline of the single loop of the localization algorithm that is executed for each LiDAR scan point.

Given a map consisting of triangles, an initial robot position, and readings from IMU and LiDAR scan data, the task is to track the location of the robot as it moves through the map. We use a modified EKF procedure to accomplish this task. The outline of the algorithm is shown in Figure 1. It is executed by applying the following steps to each scan point received from the LiDAR scanner:

1. prediction of the robot’s position and orientation based on inertial navigation sensors and prior knowledge concerning localization and
2. updating of the robot’s position relative to the closest triangle in the map to the LiDAR scan point.

The prediction step in our algorithm is straightforward. The predictions are computed based on accelerometer and gyroscope data from the 7-element state vector representing the robot’s position and orientation. The update step is more complex. It is performed in two phases. In the first phase, the algorithm uses the predicted pose of the robot and transforms the scan point from the scanner frame into a global frame. Subsequently, the algorithm finds the nearest triangle in the triangular mesh map to this single scan point (in the global frame) using the map search algorithm [68]. Next, the value of the distance between this closest triangle and the scan point is calculated. This value is treated as a measurement residual (the innovation) to calculate the updated state vector. These two steps are described in detail in the following two subsections.

#### Map Search

**Figure 2:**
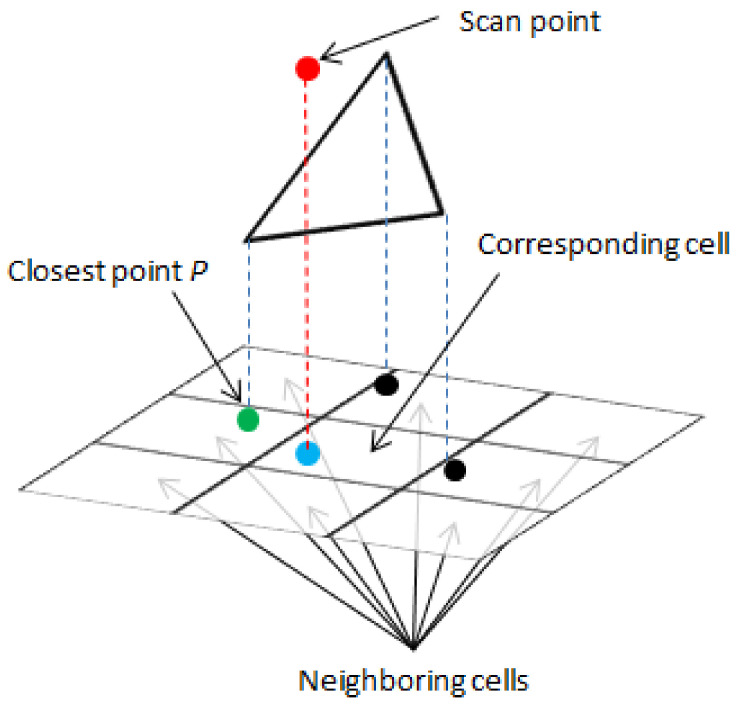
Example of a neighbor search on a grid when there is no triangle with a vertex in the queried cell, meaning that three 8-connected neighbors must be traversed.

For the algorithm to succeed, it is necessary to quickly and accurately find the closest triangle to the scan point. To do so, we use the structure described below to store the triangular mesh, which allows us to find the closest point in the 3D triangular mesh effectively. In this structure, each point of the 3D triangular mesh stores information about the adjacent triangle edges, and each edge stores information about its endpoints and faces. Additionally, we create a 2D grid corresponding to the 3D triangular mesh by projecting the vertices of the 3D mesh onto a 2D XY surface. This 2D surface is divided into cells of constant size that form a 2D grid. The points inside each cell are stored in a k-d tree to accelerate the search algorithm. This structure requires a relatively large amount of space to store the 3D mesh but accelerates the algorithm for finding the closest triangle to a given scan point. The algorithm proceeds as follows:

1. find the projection of the scan point (the red dot in Figure 2) onto the 2D surface (the blue dot in Figure 2),
2. find the corresponding cell on the 2D surface,
3. find the eight neighboring cells,
4. find all mesh vertices within a radius R of point P,
5. find the corresponding vertices of the 3D triangular mesh, and
6. for each point within radius R, take all triangles that have point P as a vertex and choose the closest among them.

This algorithm is illustrated in Figure 2. The pseudo-code for the algorithm is shown in Algorithm 1. It should be noted that the physical implementation slightly differs from the code given here—a simple optimization method is applied to avoid traversing the same triangles multiple times, but since our final solution is optimized to reduce branch divergence during parallel execution, including this optimization would introduce a certain level of confusion in understanding the algorithm, and thus, it has been omitted.
**Algorithm 1:** triangleSearch method.
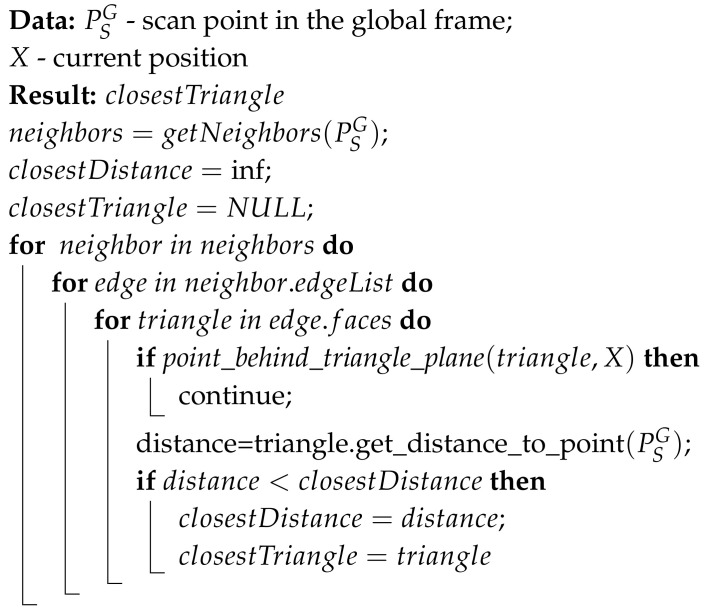


### 2.2. Ekf Procedure

After successful transformation into the triangular map domain, a modified version of the EKF procedure from [67] is applied. The implemented solution exploits a motion model based on the following equation: $X_{t}=f\left(X_{t-1},u_{t},s_{f_{t}}\right)$, with $X={\left[x,y,z,q_{w},q_{x},q_{y},q_{z}\right]}^{T}$, where *x*, *y*, and *z* represent the position of the robot; $q_{w}$, $q_{x}$, $q_{y}$, and $q_{z}$ represent the normalized quaternion *q* of rotation relative to the map; and $s_{f_{t}}$ is a zero-mean Gaussian process noise vector with covariance *Q*. The process of prediction based on IMU readings requires inputs *T* and *u*, where *T* is the time elapsed since the last prediction and $u=\left[\begin{array}{c} v\\ \omega \end{array}\right]$ is the input vector, with $v={\left[v_{x},v_{y},v_{z}\right]}^{T}$ being a velocity vector and $\omega ={\left[\omega_{x},\omega_{y},\omega_{z}\right]}^{T}$ being an angular velocity vector in the local coordinate system. The motion model after the expansion is given in Equation (1):

(1) $$X_{t}=X_{t-1}+\left[\begin{array}{c} R^{R}\left(q_{t-1}\right)\cdot v_{t}\\ 0.5{\left(\omega_{t}\right)}_{q}⊛q_{t-1} \end{array}\right]\cdot T+s_{f_{t}},$$

where $R^{R}\left(q_{t-1}\right)$ denotes a rotation matrix that depends on the quaternion $q_{t-1}$, ${\left(\omega_{t}\right)}_{q}$ is a quaternion with a zero-value scalar part and $\omega_{t}$ as the vector part, and the symbol ‘⊛’ represents quaternion multiplication.

Based on this motion model, we formulate the prediction equations as follows:

(2) $${\hat{X}}_{t|t-1}={\hat{X}}_{t-1|t-1}+\left[\begin{array}{c} R^{R}\left({\hat{q}}_{t-1}\right)\cdot v_{t}\\ 0.5{\left(\omega_{t}\right)}_{q}⊛q_{t-1} \end{array}\right]\cdot T.$$

The following equation then specifies the covariance matrix $P_{t|t-1}$ for the state ${\hat{X}}_{t|t-1}$:

(3) $$P_{t|t-1}=F_{t}\cdot P_{t-1|t-1}F_{t}^{T}+Q_{t},$$

where $Q_{t}$ is a process noise covariance expressed as $Q_{t}=G_{t}\cdot V_{t}\cdot G_{t}^{T}$, with $V_{t}$ being the covariance matrix of the measured velocity, $F_{t}=\frac{\partial f}{\partial X_{t-1}}$ and $G_{t}=\frac{\partial f}{\partial u_{t}}$.

**Figure 3:**
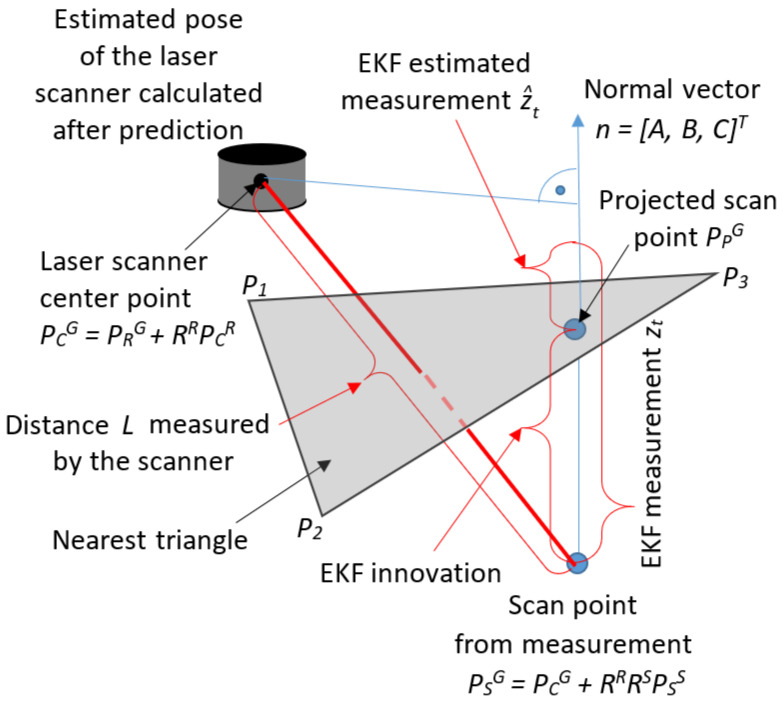
Innovation in the EKF (in this paper, we use the following notation for representing points: the subscript represents the name of the point and the superscript indicates the coordinate system: G—global, R—robot, or S—scanner).

The algorithm assumes that the correction to the estimate of the state vector is measured in the direction orthogonal to the terrain plane (see Figure 3).

The estimate of the robot’s pose obtained in the prediction phase is expressed by a vector ${\hat{X}}_{t|t-1}$, which contains the position $P_{R}^{G}={\left[\hat{x},\hat{y},\hat{z}\right]}^{T}$ and orientation $R^{R}=f\left({\hat{q}}_{w},{\hat{q}}_{x},{\hat{q}}_{y},{\hat{q}}_{z}\right)$ the robot. Based on this vector, we calculate the coordinates of the estimated scan point in the global frame.

The estimated scan point is $P_{S}^{G}={\left[{\hat{x}}_{S}^{G},{\hat{y}}_{S}^{G},{\hat{z}}_{S}^{G}\right]}^{T}=P_{R}^{G}+R^{R}\left(R^{S}P_{S}^{S}+P_{C}^{R}\right)$, where $R^{R}=f\left({\hat{q}}_{w},{\hat{q}}_{x},{\hat{q}}_{y},{\hat{q}}_{z}\right)$ is the orientation matrix of the robot with respect to the global frame, $R^{S}$ is the constant orientation matrix of the scanner with respect to the robot frame, $P_{C}^{R}$ is the position of the scanner with respect to the robot frame, and $P_{S}^{S}={\left[{\hat{x}}_{S}^{S},{\hat{y}}_{S}^{S},{\hat{z}}_{S}^{S}\right]}^{T}$ is the scan point (scanner measurement) expressed in the scanner frame.

Subsequently, we run the map search algorithm to determine the closest triangle to the scan point. This triangle represents the terrain surface, based on the coordinates of its three points $P_{1}$, $P_{2}$, and $P_{3}$, ordered in accordance with the right-hand rule, and the covariance matrix $P_{map}$ of these three vertices. The plane created by these three points is expressed as follows:

(4) $$\pi_{P_{1}P_{2}P_{3}}:Ax+By+Cz+D=0,$$

where $n={\left[A,B,C\right]}^{T}=(P2P3\times P2P1)/|P2P3\times P2P1|$ is the normal vector and $D=-n^{T}P_{2}$.

The measurement model expresses the distance measured by the scanner, projected onto the direction orthogonal to the nearest triangle to the scan point:

(5) $$z_{t}=n^{T}\left(P_{C}^{G}-P_{S}^{G}\right)=h\left(X_{t},P_{S}^{S},P_{1},P_{2},P_{3}\right)+s_{h_{t}},$$

where $P_{S}^{G}={\left[x_{S}^{G},y_{S}^{G},z_{S}^{G}\right]}^{T}=f\left(X_{t},P_{S}^{S}\right)$ is the scan point transformed into the global frame and *n* is the normal vector of the terrain plane calculated based on the nearest-triangle points, $P_{1}$, $P_{2}$, and $P_{3}$, and the center of the scanner, $P_{C}^{G}=P_{R}^{G}+R^{R}P_{C}^{R}$. The parameter $s_{h_{t}}$ represents the noise in the measurement model, including the the scanner’s measurement noise, the map noise, and the noise in the robot’s estimated position.

The estimated measurement expresses the distance between the scanner center $P_{C}^{G}$ and the scan point projected onto the terrain plane, $P_{P}^{G}={\left[x_{P}^{G},y_{P}^{G},z_{P}^{G}\right]}^{T}$, as measured in the normal direction:

(6) $${\hat{z}}_{t}=n^{T}\left(P_{C}^{G}-P_{P}^{G}\right)=n^{T}P_{C}^{G}+D.$$

The measurement residual (the innovation value) used to compute the updated pose of the robot is the difference between the measurement and its estimated value. It represents the distance from the scan point to the plane of the nearest triangle, as measured in the normal direction (see Figure 3):

$$i_{t}=z_{t}-{\hat{z}}_{t}=n^{T}\left(P_{P}^{G}-P_{S}^{G}\right)=-n^{T}P_{S}^{G}-D$$

The innovation covariance *S* can be expressed following the error propagation rule, under the assumption that the scanner measurement, the estimated pose of the robot, and the estimated map parameters are independent:

(7) $$S_{t}=HPH^{T}+N_{scan}P_{scan}N_{scan}^{T}+N_{map}P_{map}N_{map}^{T},$$

where *H*, $N_{scan}$ and $N_{map}$ are Jacobian matrices of the forms $H=\frac{\partial h}{\partial X_{t}}$, $N_{scan}=\frac{\partial h}{\partial P_{S}^{S}}$, and $N_{map}=\frac{\partial h}{\partial \left(P_{1},P_{2},P_{3}\right)}$, respectively, and *P*, $P_{scan}$ and $P_{map}$ are the covariance matrices of the robot state, the scan point in the local frame and the triangle vertices ($P_{1}$, $P_{2}$, and $P_{3}$), respectively.

Based on the innovation covariance *S*, we calculate the Kalman gain as follows:

(8) $$K_{t}=PH^{T}S_{t}^{-1}.$$

Finally, we calculate the updated robot pose:

(9) $${\hat{X}}_{t|t}={\hat{X}}_{t|t-1}+K_{t}i_{t}.$$

Since the innovation covariance matrix *S* has dimensions of 1×1, the execution of the EKF procedure is very fast (it is not necessary to perform a time-consuming matrix inversion operation), and it is possible to perform this correction procedure after every single laser scanner measurement.

## 3. Experimental

This section describes the development of our final algorithm and is divided into three sections:

1. the first section introduces the *serial algorithm*, where our initial solution to the problem is discussed;
2. the second section introduces the *parallel algorithm*, where our method inspired by [69] is discussed; and
3. the third section introduces the *parallel-serial algorithm*, where our innovative approach for satisfying given time constraints is detailed.

For simplicity of presentation, in all of the presented algorithms, the timestamp *T* is assumed to be constant over time. To remove outliers from our scans, we calculate the Mahalanobis distance of measurement innovation. If the Mahalanobis distance exceeds the experimentally chosen threshold, we drop the scan point. The threshold value should be selected depending on scanner parameters and the characteristics of the environment.

### 3.1. Serial Algorithm

In the serial localization algorithm, each scan point is processed sequentially, which means that the following steps are executed consecutively for each scan point:

- prediction;
- map search;
- calculation of the innovation, derivative, and Kalman gain; and
- updating.

**Figure 4:**
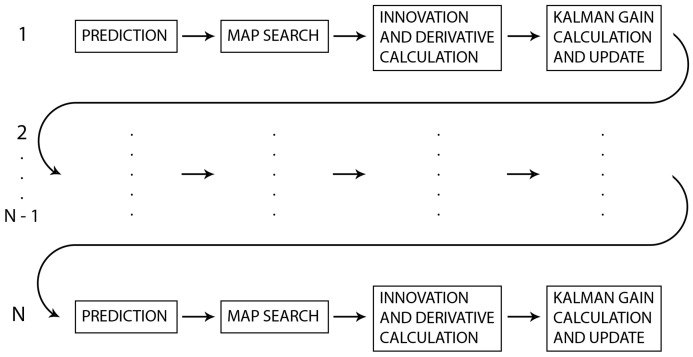
Serial algorithm.

For the case of *N* consecutive scan points, the serial localization algorithm is described in Algorithm 2 and illustrated in Figure 4.
**Algorithm 2:** Serial Kalman method.
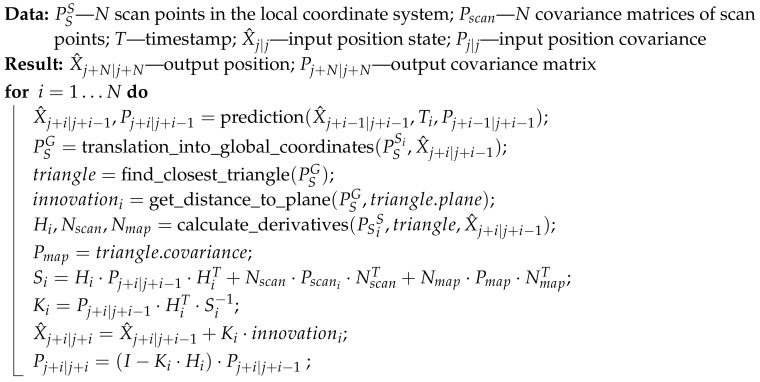


This approach results in better localization than state-of-the-art algorithms in terms of accuracy but lags behind in terms of speed. As a result, it is impossible to use this approach in real-time applications due to time constraints.

### 3.2. Parallel Algorithm

To reduce the computation time, we have developed a parallel algorithm inspired by [69]. This EKF-based method has been widely used and has many modifications and extensions [70,71]. This method uses all observed landmarks corresponding to the current timestamp (more precisely, a finite period of time, since the point cloud required for landmark extraction is captured over a finite period of time) for correction of the pose vector in a single iteration of the EKF procedure. We modify this approach by considering all scan points collected within a given period of time as landmarks and using them in a single EKF iteration to correct the pose vector. In our parallel algorithm, the steps of

**Figure 5:**
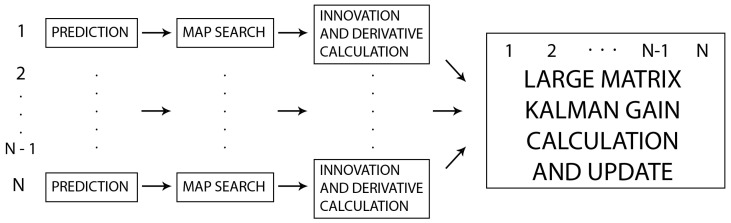
Parallel algorithm.

- prediction,
- map search, and
- the calculation of the innovation and derivative

are computed in parallel. For each *N* results of parallel computations, we execute a single Kalman gain calculation and update process. We treat each output from the parallel algorithm as a separate input for the Kalman gain, which results in the multiplication and inversion of *N*-by-*N* matrices. Operations on such matrices can also be parallelized by using CUDA parallel matrix libraries. This leads to the parallel algorithm, which is detailed in the form of pseudo-code in Algorithm 3 and illustrated in Figure 5.
**Algorithm 3:** Parallel algorithm.
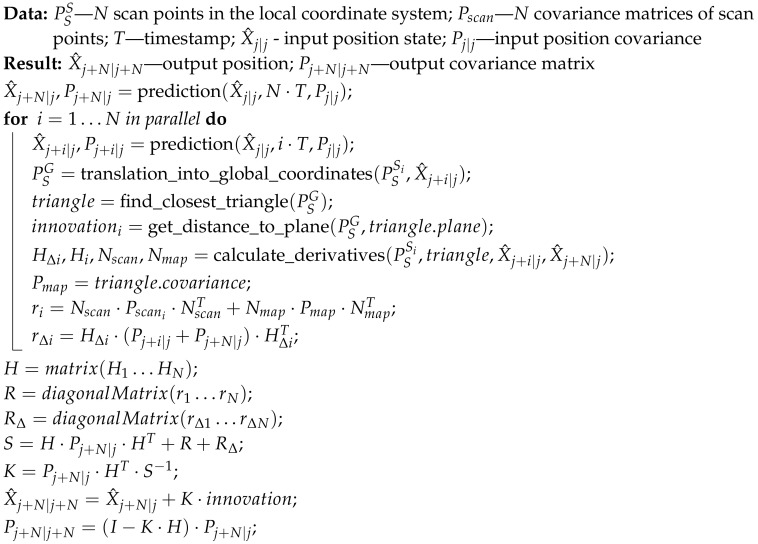


The main flaw in this algorithm is that after each parallel iteration, a single *N*-by-*N* dense matrix needs to be inverted, which is computationally costly. We have tested the algorithm using two state-of-the-art GPU matrix libraries, namely cuBLAS 10.0 and MAGMA, using small *N* values. Our test results lead us to conclude that even with the use of state-of-the-art parallel libraries, the algorithm execution times are still far too long for real-time applications.

### 3.3. Parallel-Serial Algorithm

**Figure 6:**
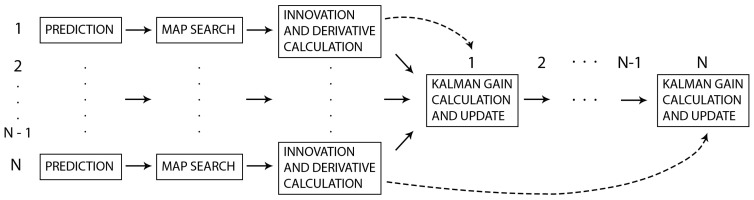
Parallel-serial algorithm.

To overcome the parallel algorithm’s limitations, we have developed a parallel-serial localization algorithm that combines the serial algorithm described in Section 3.1 with the parallel algorithm described in Section 3.2. In this algorithm, parallel computations are performed following the algorithm in Section 3.2, except that the position covariance is not recalculated in the prediction step. After the parallel part finishes, the Kalman gain is calculated for each step consecutively, as in Section 3.1, but with a certain enhancement. The difference is that at the beginning of each serial step, the prediction based on the position from the previous step is compared with the position from parallel prediction. This vector is multiplied by the derivative from the *i*-th parallel iteration and subtracted from the innovation from the *i*-th parallel iteration. The rest of the algorithm follows this serial Kalman enhancement. The algorithm is summarized in Figure 6, and the pseudo-code for the parallel-serial Kalman computations is given in Algorithm 4.
**Algorithm 4:** Parallel-serial Kalman method.
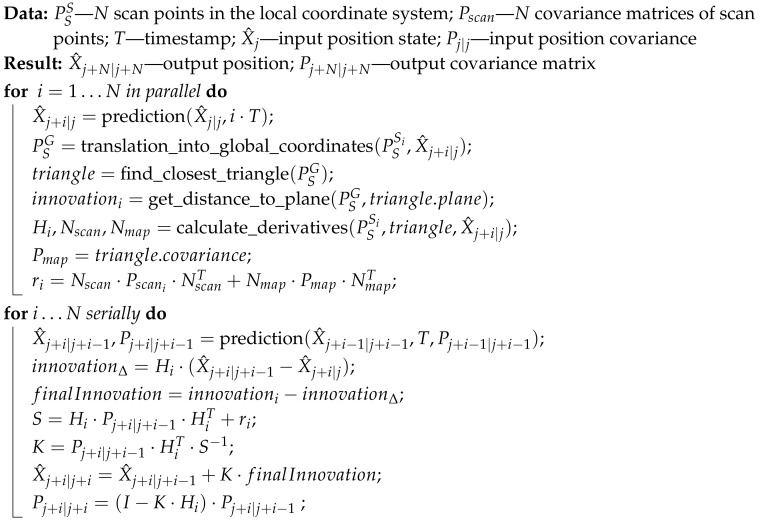


An important note about the algorithm introduced here is that in the serial part, the position and covariance matrix from the previous iteration are used in the current iteration and are compared against the supposed position from the parallel part to estimate the error of the current measurement. It should also be noted that prediction is performed twice, which implies that this algorithm requires more computation than the serial method does and that the serial part is computationally costly. In fact, the estimation of the closest triangle is the calculation that exerts the greatest impact on the computation speed of the whole procedure, mostly due to the need to find the exact distance from the point to the triangle. The duplication between the serial and parallel parts can be deemed computationally irrelevant compared to other key parts of the procedure and thus is omitted in further analysis.

## 4. Calculation

To verify the computation speed and accuracy of the parallel-serial algorithm introduced here, we implemented it in CUDA and executed it on two different computers installed on our robot. The robot was navigating in two test environments: a mine gallery and a cave.

### 4.1. Hardware

The parallel-serial algorithm was executed using two different computers:

- an NVIDIA Jetson TX2 and
- an NVIDIA Jetson Xavier AGX.

These two computers were installed on the robot, and experiments were performed under real-world conditions. All the calculations and estimations considered in this paper were performed using this hardware. The robot was equipped with a VLP 32C Velodyne scanner. The LiDAR head rotation speed was approximately 600 rpm, and the frequency of the received scan points was downsampled to 300 kHz.

### 4.2. Testing Environments

**Figure 7:**
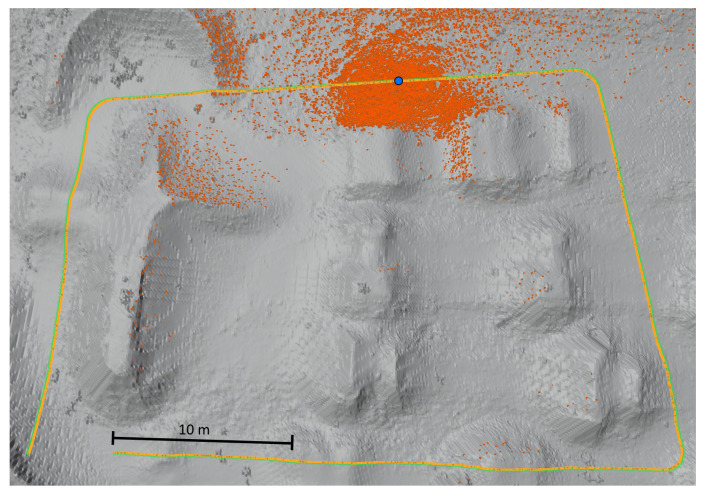
Horizontal cross-section of the point cloud from the mine gallery. The figure shows: the reference scan (gray surface), the measured path of the robot (yellow curve), reference path (green curve), 50,000 scan points captured in 0.1 s during localization (orange dots), localization of the robot when LiDAR was capturing measurement points (blue point).

**Figure 8:**
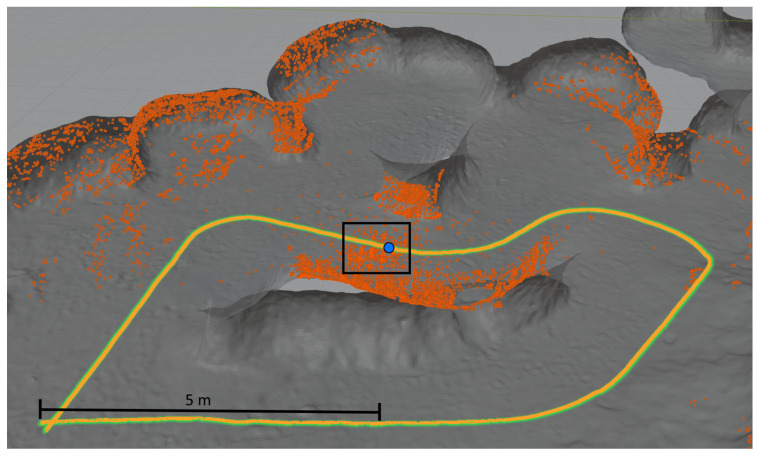
Horizontal cross-section of the point cloud from the cave. The figure shows the reference scan (gray surface), the measured path of the robot (yellow curve), reference path (green line), 50,000 scan points captured in 0.1 s during localization (orange dots), localization of the robot when LiDAR was capturing measurement points (blue point), the approximate location of the zoomed view shown in Figure 9 (black rectangle).

The parallel-serial localization algorithm was tested in two environments: a mine gallery and a cave. Triangular mesh maps for both testing environments were generated from point clouds. These point clouds were captured using reference stationary FARO scanners from arbitrarily chosen fixed locations. We used an ICP-based scan matching algorithm to register all point clouds from each environment into a common frame. Subsequently, we generated a triangular mesh map from the overall point cloud for each testing environment. These triangular mesh maps were treated as reference maps for the localization algorithm described in Section 3.3. Based on the reference point clouds, we also generated Gaussian mixture maps (2.5D surfel-based maps) to be used as the input for the localization method of [66], which was chosen as the reference method. Figure 7 shows the horizontal cross-section of the point cloud (gray surface) obtained for the mine gallery together with the reference path (green curve) and the measured path (yellow curve). We have compared the algorithm using a virtual reference path to avoid the errors introduced by the reference path measurement and robot control errors. The robot movement and LiDAR measurements were obtained by selecting scan points from the dense, static point clouds of the cave and the mine gallery captured using a static scanner. The point clouds were obtained by merging point clouds from several static scans. IMU output was also simulated by adding Gaussian noise to the ground-truth localization of the robot. As a result, our reference path can be assumed to have perfect accuracy. We want to emphasize that it was performed just for the purpose of the comparison of our algorithm to the state of the art algorithms, and the localization system runs on the hardware mounted on the robot. The measured path overlaps with the reference path due to the high accuracy of the localization process. The orange dots represent 50,000 scan points captured in 0.1 s, which corresponds to 20 cm distance traversed by the robot, recorded at an arbitrary point in time during the localization process. The blue point represents the localization of the robot when the LiDAR was capturing measurement points (marked with orange color). Total path length in this experiment: 121.916 m, total time it took the robot to traverse it: 60.9 s. An analogous visualization is presented in Figure 8 for the cave environment. All colours on both figures are the same. The only difference is the total path length which in case of cave is 45,294 m. The total time it took the robot to traverse it is 22.6 s. The map resolution is expressed in terms of the edge length of the triangles. For the mine gallery (Figure 7), the triangles had an edge length of approximately 20 cm. For the cave (Figure 8), the mesh was much denser, with triangle edges of approximately 3 cm on average. In general, it is possible to use edge lengths from 1 cm to 30 cm, and the recommended resolution depends on the curvature of the measured surroundings as well as the accuracy of the LiDAR scanner—the error of the created map should be no greater than the error of the LiDAR measurements.

**Figure 9:**
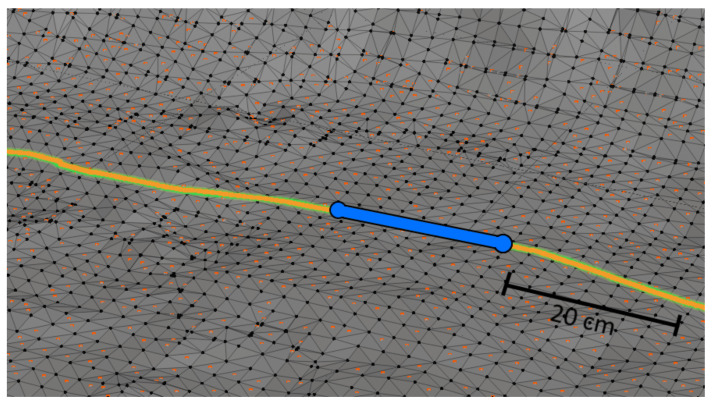
Fragment of a triangular mesh. The figure shows: vertices of the triangle mesh (black dots), scan points captured in 0.1 s during localization (orange dots), robot localization (yellow curve), reference path (green curve), localization of the robot when the LiDAR was capturing measurement points (blue line).

For both testing environments, we generated reference triangular mesh maps. Due to space limitations, we cannot present the complete triangular meshes for both testing environments here; instead, we show only a small fragment of a triangular mesh in Figure 9. In this figure, the black dots represent the vertices of the triangular mesh. The orange dots represent the scan points used for robot localization. The corresponding robot localization results are marked in yellow. The blue line represents the robot’s localization when the LiDAR was capturing measurement points marked with orange color. The reference path for the localization of the robot is also marked using a green line, but this line is barely visible because the yellow line overlaps it. The parameters of both test datasets are summarized in Table 1. The table contains the reference tracks lengths, the size of the point cloud, corresponding triangle meshes, and Gaussian mixture model (GMM) map for both tracks in the cave and the mine gallery. The cave dataset is available here https://3dskaner.p.lodz.pl/udostepnione-dane-projektowe/ for download. The mine gallery dataset is not available due to licensing restrictions.

## 5. Results

The localization algorithm introduced in Section 3.3 was tested using the hardware described in Section 4.1 in the testing environments detailed in Section 4.2. As the reference algorithm, we chose the method presented in [66] because it is considered a state-of-the-art algorithm for 3D localization. We compared our parallel-serial algorithm with this reference algorithm using the same input data. We used the following parameters for the comparison:

- scanner angular speed: 600 rpm,
- scanner sampling frequency: 300,000 points per second,
- max. scan size per single registration: 10,000 points,
- linear resolution of registration: 1 mm,
- heading angular resolution of registration: 0.01 deg,
- innovation permissible value in EKF linear part: 0.25 m.

**Figure 10:**
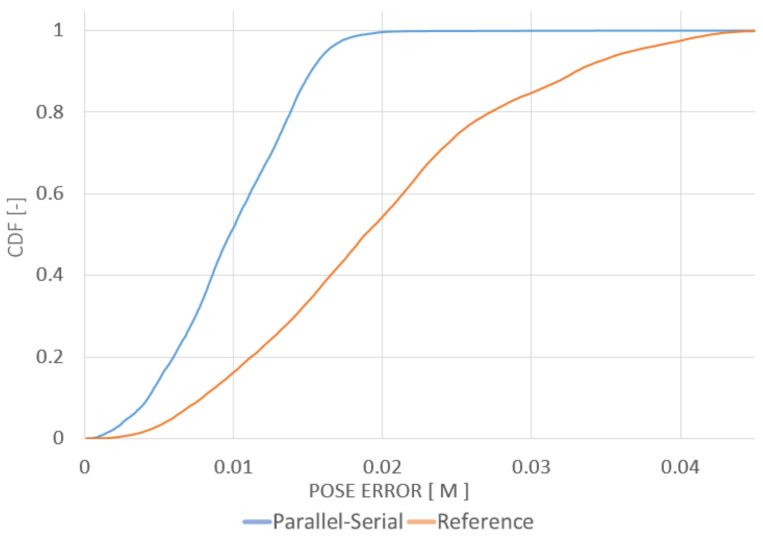
CDF of the position error relative to the ground-truth path with parallel-serial localization on the path projected in Figure 7. The orange line shows the reference localization algorithm results from [66], while the blue line shows the results of our method.

**Figure 11:**
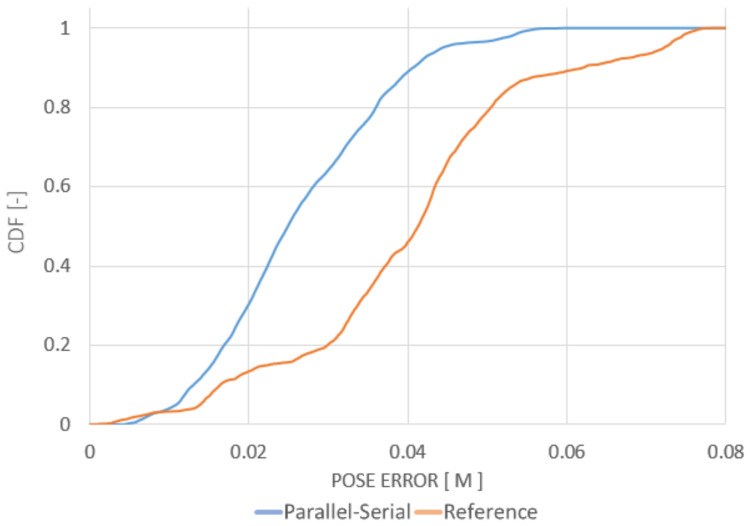
CDF of the position error relative to the ground-truth path with parallel-serial localization on the path projected in Figure 8. The orange line shows the reference localization algorithm results from [66], while the blue line shows the results of our method.

The maps created *a priori* for the localization tests were built using the same point clouds registered into a common frame. The final paths of the robot obtained using the localization algorithms were compared with the ground-truth path. The ground-truth path is marked with a green line in Figure 7, Figure 8 and Figure 9. The measured path is marked in yellow. The corresponding cumulative distribution functions (CDFs) of the position error for the mine gallery and cave are shown in Figure 10 and Figure 11, respectively. The RMSE error and maximum deviation for serial, parallel, parallel-serial and the reference localization algorithms are presented in Table 2. The results in these two figures and Table 2 clearly show that our parallel-serial algorithm outperforms the reference algorithms based on GMM [66] or PSD [67] in terms of localization accuracy.

## 6. Discussion

The main concern in this research is to guarantee the desired accuracy in real-time. Therefore, we also analyzed our algorithms’ time complexity using the hardware described in Section 4.1.

**Figure 12:**
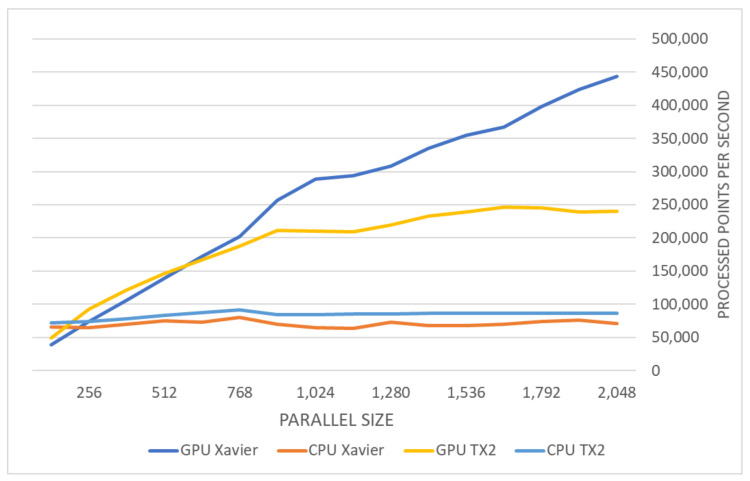
A complete comparison between: serial (CPU) EKF, parallel-serial (GPU) EKF, parallel (GPU) EKF execution for different batch sizes of up to 2048 in parallel iteration.

The results presented in Figure 12 illustrate the time complexity of the serial and parallel-serial algorithms as a function of the size of the parallel kernel (the number of points computed in parallel) on the two different computers specified in Section 4.1. Please notice that the parallel-serial algorithm introduced here outperforms both parallel and serial algorithms. This is mainly due to EKF algorithm properties. In the parallel algorithm, the Kalman gain calculation and update process require inverting a large matrix (see Figure 5), which is time-consuming even if the parallel implementation is used. To avoid it, in our parallel-serial algorithm, the position covariance is not recalculated in the prediction step. Instead, the Kalman gain is calculated for each step consecutively. At the beginning of each serial step, the prediction based on the position from the previous step is compared with the position from parallel prediction. As a result, the large matrix’s inversion is not necessary, which reduces the computational complexity of the algorithm.

**Figure 13:**
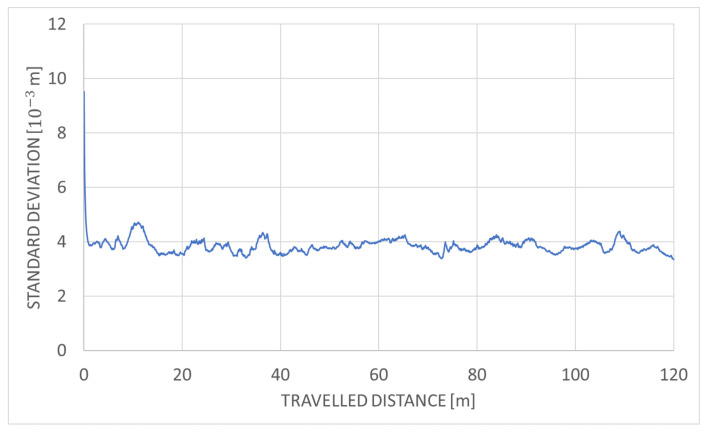
Standard deviation in function of travelled distance for parallel-serial algorithm and mine gallery test dataset.

**Figure 14:**
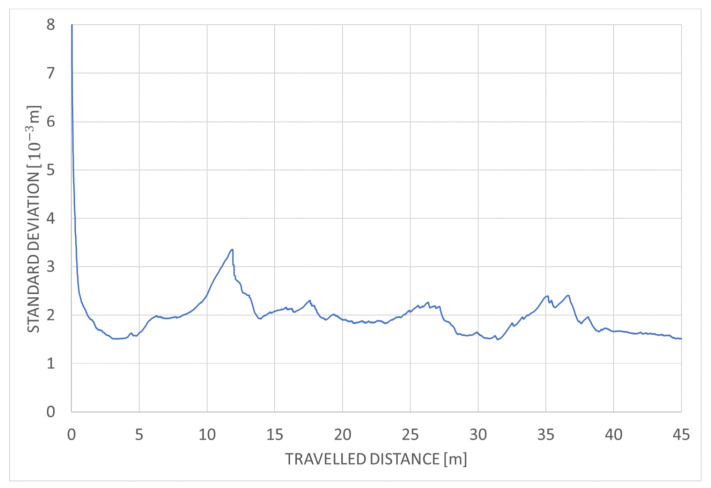
Standard deviation in function of travelled distance for parallel-serial algorithm and cave test dataset.

Figure 12 shows the overall comparison for both testing environments and highlights the difference between CPU-based and GPU-based performance. Because our LiDAR scanner captures 300 thousand points per second, a parallel implementation is required to guarantee the desired calculation speed. Table 3 summarizes the maximal performance on the tested devices. The number of points that can be used for localization can be tripled in case of Jetson TX2 and can be even six times higher when the parallel serial algorithm is executed on Jetson Xavier compared to serial implementation. The performance of GMM algorithm is much lower when executed on the same hardware. Standard deviation in function of traveled distance for the parallel-serial algorithm is shown in Figure 13 for mine gallery test dataset, and in Figure 14 for the cave dataset. The deviation shown in both figures is the square root of the trace of the position part of the covariance matrix. The error is calculated as the difference of location estimate and ground truth localization. Please note that the error value is higher than the measurement uncertainty. This is because the error is calculated relatively to the scan point, while the standard deviation is calculated relatively to the triangle mesh. Our algorithm uses triangle mesh, not a point cloud, and errors of the triangle mesh are not directly related to the measurement uncertainty determination. The value of standard deviation declines rapidly within the first several seconds (see Figure 13 and Figure 14). It reflects the initial correction of localization when the algorithm starts and is caused by the starting point’s misalignment. The value of standard deviation oscillates around 2 mm for the mine gallery dataset and approximately 4 mm for the cave dataset. It reflects the localization process performed by EKF. The difference between the standard deviation value for both datasets is caused by the difference in both LiDAR scans’ density.

To obtain optimal localization results LiDAR capturing speed should match computer processing speed. Nevertheless, the algorithm will compute the robot localization correctly, even if the computer cannot process all scan points captured by the LiDAR. In our experiments, LiDAR can capture 300 thousand points per second, while Jetson TX2 can process only 250 thousand points per second. As a result, some of the scan points are omitted by the localization algorithm because of sampling frequency mismatch. Additionally, in parallel implementation, if too many scan points are in the buffer, the oldest ones are simply omitted in the following execution of the parallel loop.

The accuracy of the localization algorithm depends on the amount of input data. That is, if the scanning speed decreases, the localization accuracy decreases. Table 4 shows how the localization accuracy of parallel-serial algorithm changes in function of LiDAR scanning speed in the cave test dataset, when the algorithm is executed on Jetson Xavier.

In the default 15 W power consumption mode, the Jetson Xavier processed over 450,000 points per second with a parallel kernel size of 2048 points, almost twice as much as the Jetson TX2 in the 15 W power consumption mode. For this kernel size, the performance on the tested embedded devices is already sufficient for application in autonomous robotics, at least in our domain—Table 5 shows an efficiency comparison between tested devices. A further increase in kernel size may additionally boost the algorithm’s performance, especially for GPUs with more processing units; to investigate this possibility, we also tested our algorithm on a PC using offline data and found that the NVIDIA RTX 2070 SUPER GPU was able to process up to 2,500,000 points per second, at the cost of almost 300 W of power consumption.

Table 5 presents a power efficiency comparison of the tested hardware. The NVIDIA Jetson Xavier outperforms the NVIDIA Jetson TX2 by almost a factor of two in terms of performance but consumes approximately 8% more energy per processed scan point.

On a single-core CPU, the serial part of the parallel-serial algorithm will never consume more than 15% of the serial algorithm’s total execution time. If we assume the worst case and approximate this contribution as 15%, then the remaining 85% can be accelerated, resulting in the following total speedup:

(10) $$\begin{array}{cc} \; & T_{p-s}^{CPU}\approx 0.15T_{s},\\ \; & T_{p-s}^{GPU}\approx \left(T_{s}-T_{p-s}^{CPU}\right)/N\approx \left(0.85\cdot T_{s}\right)/N,\\ \; & T_{p-s}\approx 0.15\cdot T_{s}+\left(0.85\cdot T_{s}\right)/N,\\ \; & T_{p-s}\approx T_{s}\cdot \left(0.15+\frac{0.85}{N}\right), \end{array}$$

where *N* is a device-dependent parallelization factor, $T_{s}$ is the estimated total execution time of our serial method, $T_{p-s}$ is the estimated total execution time of our parallel-serial method, $T_{p-s}^{CPU}$ is the estimated execution time of the CPU part of our parallel-serial method, and $T_{p-s}^{GPU}$ is the estimated execution time of the GPU part of our parallel-serial method.

The parallelization factor *N* must be determined experimentally since there are many factors influencing it, and it may vary slightly even for two identical architectures. To determine this factor, time estimates for the serial and parallel-serial procedures are needed. In our experiments, we found that the best result in terms of the factor *N* in an embedded device was achieved for the Jetson Xavier with a kernel size of 2048, for which *N* was approximately 17.

In general, the parallel-serial algorithm can easily process 250 thousand points per second, even when executed on the Jetson TX2. Because the LiDAR scanner installed on the robot captures no more 300 thousand points per second, this is a sufficient processing capacity for real-time operation. Through parallelization, the desired accuracy can be achieved in real-time.

Overall, when using the parallel-serial algorithm, the robot successfully navigated in both testing environments using a triangular mesh map as a reference. Notably, there are many factors influencing the accuracy of localization, and the maximum tolerated time interval for the parallel iterations also depends on many factors, including the velocity of the robot, the number of received scan points per second, and the accuracy of the map. Nevertheless, the results obtained here are general in the sense that the parallel-serial localization algorithm outperforms the reference algorithm in terms of accuracy while guaranteeing a similar time complexity.

It should also be noted that GPU parallelization not only accelerates the whole localization process but also dramatically reduces CPU usage, which, in turn, allows the potential for users to conduct other operations on the CPU simultaneously, e.g., Kalman fusion of data from other sensors or map expansion.

## 7. Conclusions

In this paper, we introduced a new real-time parallel-serial LiDAR-based localization algorithm for GPS-denied environments that can achieve centimeter accuracy when using a triangular mesh map as a reference for localization. It is possible even though point cloud meshing generates additional localization error. The use of triangular mesh maps instead of point clouds reduces the required storage capacity, which is essential for practical applications. We performed experiments using our mobile robot in two GPS-denied environments, namely a cave and a mine gallery. Our research has confirmed the effectiveness and feasibility of centimeter-accuracy localization in these challenging environments. The main contribution of our research is the partial parallelization of the EKF algorithm, which is essentially sequential. Importantly, however, this algorithm’s novel contributions are not limited to the parallel execution of matrix operations, which has been achieved in many publications. Our parallel-serial localization algorithm based on a triangular mesh map is general in nature and can be extended to many other applications [72].

## Figures and Tables

**Table 1:** Test datasets parameters.

Dataset	Point Cloud Size	Triangle Mesh Size	GMM Size	Total Distance Travelled
Mine gallery	360,150 kpoints 4121.5 MB	997,884 triangles 500,466 points 10.3 MB	500,466 cells 3.8 MB	121.916 m
Cave	144,060 kpoints 1648.6 MB	1,727,729 triangles 865,824 points 19.2 MB	325,254 cells 2.5 MB	45.294 m

**Table 2:** Localization accuracy of our algorithms: parallel-serial (P-S) and parallel (P), and the reference methods: GMM [66] and PSD [67].

Dataset	Mine Gallery				Cave			
Method	P-S	P	GMM	PSD	P-S	P	GMM	PSD
RMSE [cm]	1.07	2.71	3.06	3.94	2.96	4.99	4.32	6.32
Max. dev. [cm]	3.89	4.37	8.09	9.75	5.94	12.29	7.84	12.66

**Table 3:** Performance of our parallel-serial EKF algorithm in comparison with the serial EKF approach and reference method serial implementation (GMM).

Machine	Parallel-Serial EKF (GPU)	Serial EKF (CPU)	GMM (CPU)
Jetson TX2	250,000 points/s	80,000 points/s	6000 points/s
Jetson Xavier	450,000 points/s	80,000 points/s	6000 points/s

**Table 4:** Accuracy vs. measurement scan size for the cave dataset.

No. Measurements Points/s· 10^3^	300	150	75	37.5	18.75	9.375
Max. dev. [cm]	5.94	5.85	6.92	7.81	9.08	79.83
Avg error [cm]	2.75	2.76	2.77	2.83	3.66	13.75
RMSE [cm]	2.96	2.97	3.04	3.14	4.17	22.31

**Table 5:** Power efficiency of each device in terms of the average number of processed points per joule of consumed energy.

Machine	GPU Efficiency	CPU Efficiency
Jetson TX2	16.7 points/mJ	5.3 points/mJ
Jetson Xavier	15.4 points/mJ	4.1 points/mJ
PC	11.3 points/mJ	3.4 points/mJ
